# Case Report: Navigating novel therapies and systemic barriers in ovarian clear cell carcinoma through a complete response to lenvatinib, pembrolizumab, and SFRT

**DOI:** 10.3389/fonc.2025.1679554

**Published:** 2025-12-19

**Authors:** Jared Hobson, Allison E. Garda, Jonathan Ticku, Abigail Stockham

**Affiliations:** 1 Department of Radiation Oncology, Mayo Clinic, Rochester, MN, United States; 2 Department of Hematology/Oncology, Mayo Clinic Health System, La Crosse, WI, United States; 3 Department of Radiation Oncology, Mayo Clinic Health System, La Crosse, WI, United States

**Keywords:** case report, immunotherapy, ovarian cancer, radiation, SFRT, GRID, financial toxicity

## Abstract

Ovarian clear cell carcinoma (OCCC) is a rare and chemoresistant histology known for having a poor prognosis and limited treatment options. Novel therapies offer hope, but come at a significant cost, with patients facing financial toxicity, inequitable access, and systemic barriers inherent to our current cancer care infrastructure. Utilizing a case of complete pathologic response to Lenvatinib, pembrolizumab, and spatially fractionated radiotherapy (SFRT) in metastatic OCCC after overcoming numerous individual and systemic barriers, we address the challenges surrounding access and innovation in cancer treatment, while simultaneously adding evidence to support these novel therapies in OCCC treatment. While acknowledging the role of expanded access programs and right-to-try pathways, we address broader issues surrounding restrictive trial criteria, inequitable resource distribution, and cancer as a chronic disease state, asking the question: how do we develop and disseminate novel therapies while addressing toxicities in today’s healthcare system?

## Introduction

Ovarian clear cell carcinoma (OCCC) is a rare and aggressive histology known for its poor prognosis and intrinsic chemotherapy resistance, leaving patients with limited options. Novel therapies offer hope —but at significant cost, with global cancer drug spending reaching $223 billion in 2023 and projected to reach $409 billion by 2028 (1). Patients bear much of this cost, and financial toxicity has been recognized as a growing problem, disproportionately affecting low-income patients and compounding the stress of a cancer diagnosis (2, 3). For those with metastatic cancer or a poor response to conventional therapy, emerging treatments provide critical options but may be cost prohibitive, leading many to self-advocacy and crowdfunding.

Herein, we present a case of metastatic OCCC achieving a complete clinical and pathologic response to off-trial spatially fractionated radiation therapy (SFRT) and lenvatinib/pembrolizumab, despite significant personal and systemic barriers. This case highlights the potential synergistic efficacy of these therapies, and the broader financial and logistical challenges patients face in accessing innovative care.

## Consent

Patient informed consent was obtained for the publication of this case and commentary.

## Case presentation

**Figure 1:**
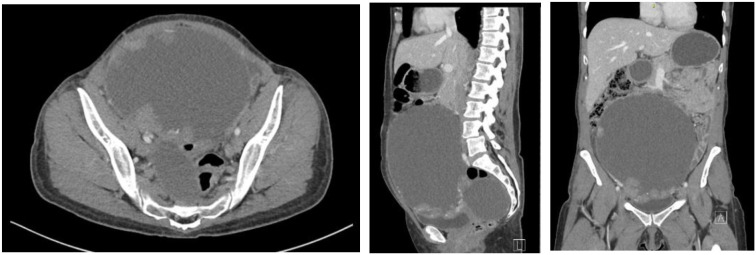
CT AP with contrast 2/4/2021: 18.4cm cystic mass arising from the right adnexa, with an additional 8.8cm cystic lesion in the posterior right pelvis.

A 49-year-old female with no significant medical history noted a lump in her right lower abdomen in August 2020 but delayed evaluation due to family emergencies, including her spouse’s death. By February 2021, the mass had enlarged, accompanied by weight loss. Physical examination noted a large midline mass resembling a gravid uterus measuring 26 cm. Ultrasound showed a 17 cm cystic and solid right pelvic wall mass presumed to arise from the right adnexa, with an additional 8 cm mass within the right ovary. CBC, CMP, and chest x-ray were unremarkable aside from hypercalcemia, and CA-125 was elevated at 213 U/mL. Computed tomography (CT) of the abdomen and pelvis (AP) confirmed two large pelvic masses with no definite metastases (**Figure 1**). Cytoreductive surgery was performed 2/5/2021, with significant endometriosis, adhesions, and bilateral ureteral scarring resulting in a modified radical hysterectomy with bilateral salpingo-oophorectomy. Final pathology revealed FIGO Stage IIIB high-grade clear cell carcinoma with severe endometriosis, pT3b pN0, with positive bladder peritoneum and Morrison’s pouch involvement. Myriad genetic testing was negative, with no clinically significant germline mutations, and somatic profiling negative for BRCA1/2, GIS negative. She completed six cycles of carboplatin/paclitaxel on 6/17/2021, with no imaging evidence of disease and CA-125 stable at 13 U/mL.

**Figure 2:**
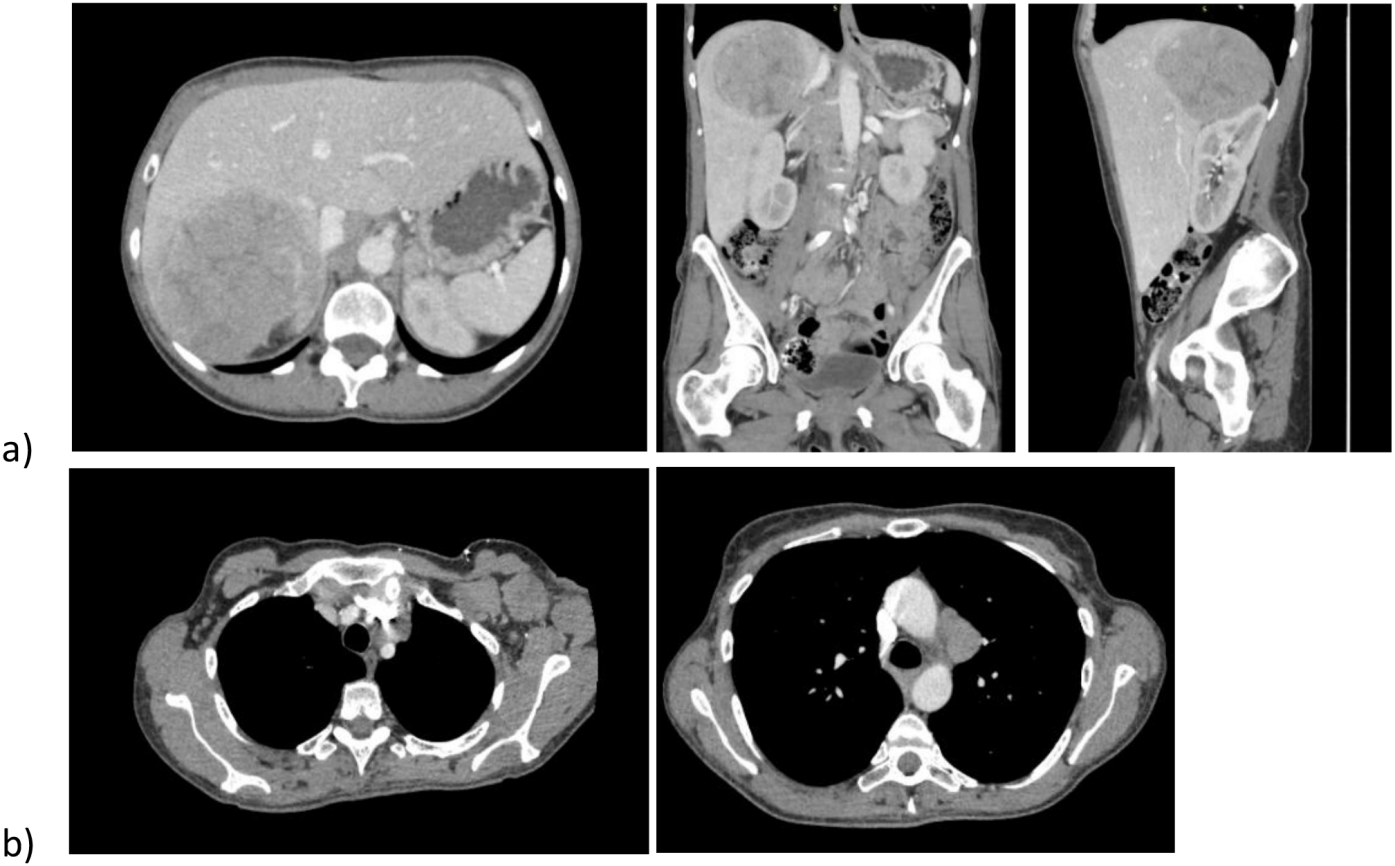
**(A)** CT AP with contrast 7/15/2022: new right hepatic mass up to 10 cm with new extensive retroperitoneal, periaortic, and pericaval adenopathy. **(B)** CT Chest 7/15/2022: new left axillary (up to 3.9 cm) and mediastinal adenopathy (up to 3.4 cm).

Approximately 1 year later, she reported unintentional weight loss, nausea, and malaise. Repeat CT chest, abdomen, and pelvis (CAP) on 7/15/2022 showed new retroperitoneal, mediastinal, and left axillary nodal metastases and a 10 cm right hepatic lesion (**Figure 2**). Labs showed CA-125 = 36 U/mL, hemoglobin 9.7 g/dL, and hypercalcemia >16 mg/dL requiring hospitalization. She began carboplatin/gemcitabine on 8/12/2022, complicated by a DVT requiring Eliquis and persistent hypercalcemia managed with Zometa and Denosumab. CA-125 peaked at 128 U/mL before declining to 74. CT CAP 10/2022 showed stable adenopathy but hepatic lesion progression, prompting addition of bevacizumab, and a small incidental PE. Interval CT CAP on 1/29/2023 showed stable adenopathy and PE resolution; however, the hepatic metastasis had grown to 12.6 cm. Hepatectomy was considered but deemed of unclear benefit.

**Figure 3:**
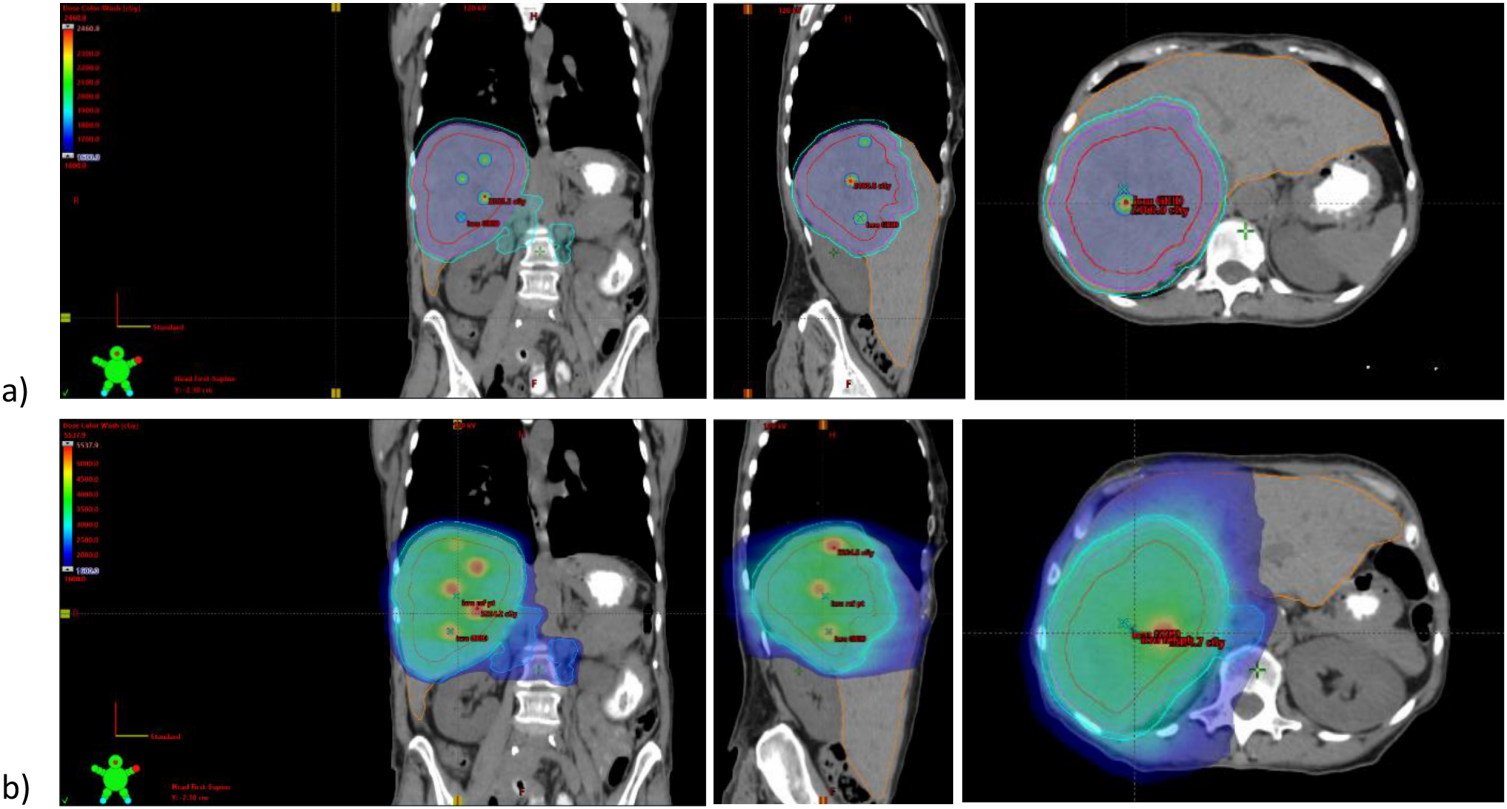
Radiation plan. **(A)** SFRT 20 Gy in 1 fraction to 5 spheres placed within GTV, delivered 4/4/2023. Dmax 24.61 Gy. **(B)** 30 Gy in 5 fractions to right hepatic mass PTV. 20 Gy in 5 fractions to involved coplanar retroperitoneal nodes PTV, delivered 4/5/2023 – 4/11/2023. V95% = 100% for targets. Liver CV21Gy = 1306cc.

At this time, alternate systemic therapy, targeted radioembolization, and radiotherapy were discussed, but the patient sought additional surgical opinions, stating, *“I will have that surgery, even if I have to go [elsewhere] or out of the country. I know for a fact it would help. Maybe I’ll have five months…. but I’m going to fight.”* Outside tumor board again recommended against liver resection and she met with radiation oncology but declined radiation, fearing ineligibility for future surgery. Clinical trials, including phase II trials with ipilimumab/nivolumab and lenvatinib/pembrolizumab at outside institutions were discussed. Interim imaging on 3/15/2023 showed hepatic mass progression to 13.5 cm, and she elected to undergo radiation while seeking lenvatinib/pembrolizumab trial enrollment. Given the tumor size, adjacent retroperitoneal nodes, and proximity to small bowel limiting dose, SFRT was pursued to optimize response while limiting toxicity. She received volumetric arc radiotherapy (VMAT) SFRT 20 Gy in 1 fraction forming 5 spheres within the right hepatic mass on 4/4/2023; planned per Grams et al. with 1.5 cm spheres placed within the gross tumor volume (GTV) with at least a 3 cm center-to-center separation and at least 1 cm from sphere edge to any organ at risk (OAR) (4). This was followed by 30 Gy in 5 fractions to an internal target volume (ITV) and 5 mm planning target volume (PTV) from the hepatic mass with a lower 20 Gy dose to nearby enlarged retroperitoneal nodes delivered 4/5/2023 - 4/11/2023 (**Figure 3**). Radiation was tolerated well with grade 2 nausea managed expectantly and no significant changes to liver function to date. She qualified for the lenvatinib/pembrolizumab trial (ID 21-739) but was deemed ineligible due to an incidental PE on CT 5/2/2023.

Denied trial access, she continued carboplatin/gemcitabine/bevacizumab while seeking insurance authorization for lenvatinib/pembrolizumab off-trial. Authorization was granted following initial denial and appeal. Meanwhile, despite prior auto-approval, radiation coverage was denied post-treatment, cited as experimental, requiring a year of appeals and financial navigation. She started lenvatinib 20 mg daily and pembrolizumab 200 mg Q3 weeks on 5/24/2023, with improvements in fatigue and abdominal pain. Imaging in August and November 2023 showed a reduction in the hepatic mass to 8.2cm with stable adenopathy. Calcium levels decreased and CA-125 stabilized at 16 U/mL. Following the new year her employer-sponsored health insurance switched companies. Although pembrolizumab was initially approved (February 2024), she received a denial letter again citing expiremental treatment, delaying treatment and requiring numerous appeals, letters, phone calls and even state congressional involvement to restore coverage.

**Figure 4:**
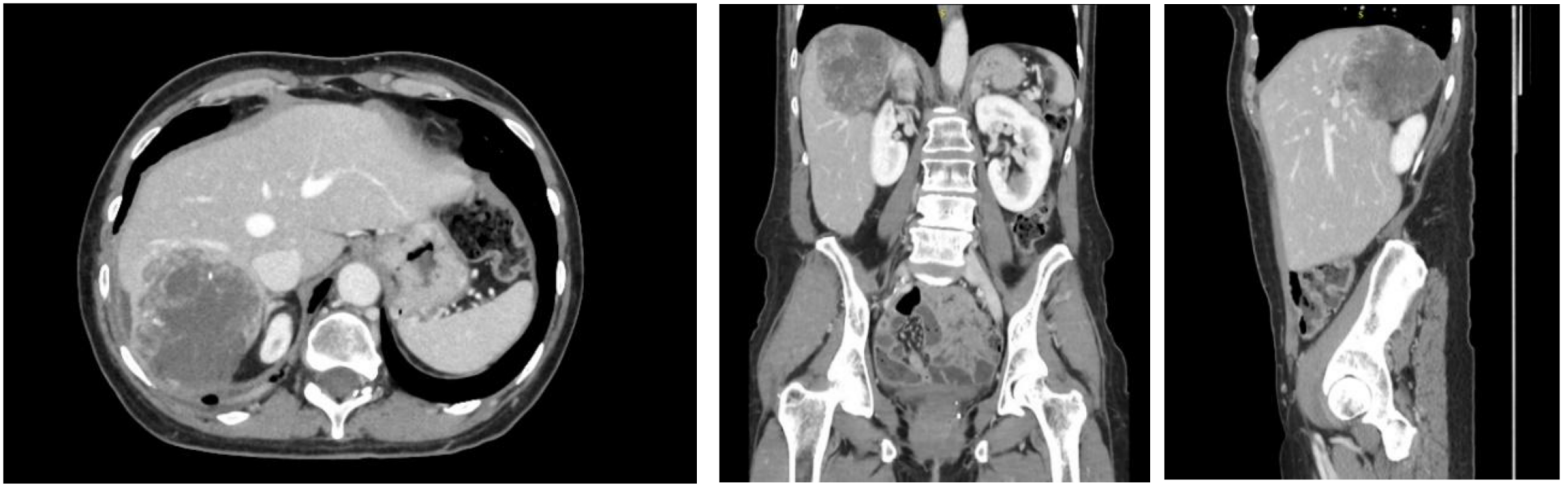
CT AP with contrast 5/29/2024: Decreased hepatic mass now 7.7 x 6.3 cm. Stable upper retroperitoneal adenopathy.

**Figure 5:**
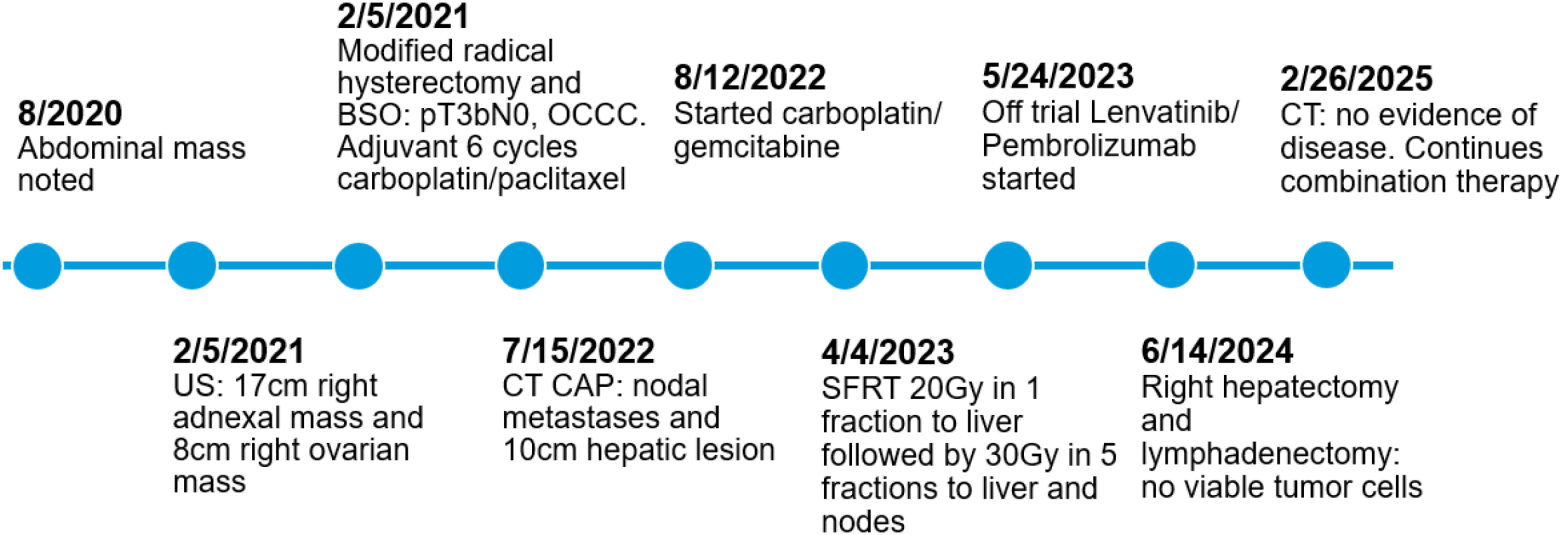
Timeline of events.

Subsequent MRI Abdomen on 4/26/2024 showed hepatic lesion shrinkage (8.0 cm) with stable adenopathy aside from a slightly increased peri-aortic node and a chronic IVC thrombus. Reporting persistent abdominal pain and hypercalcemia, local therapy was reconsidered. Repeat CT CAP on 5/29/2024 confirmed MRI findings with no new disease (**Figure 4**) and following multidisciplinary discussion, exploratory laparotomy with right hepatectomy, cholecystectomy, and radical retroperitoneal lymphadenectomy was performed 6/14/2024. Final pathology demonstrated a complete pathologic response with no viable tumor cells. At publication, she continues lenvatinib/pembrolizumab with intermittent holds for immunotherapy-related colitis, with stable CA-125 at 17 U/mL and recent CT 10/15/2025 showing no evidence of disease. A summary timeline of key clinical events and treatments can be seen below in **Figure 5**.

## Discussion

This case adds supporting evidence for lenvatinib and pembrolizumab in advanced ovarian cancer and highlights SFRT’s potential. Overcoming denials and system challenges, her case exemplifies the multifaceted burdens of cancer care, and begs the question: how do we develop and disseminate novel therapies while addressing toxicities in today’s healthcare system?

### Ovarian clear cell carcinoma

With a 5-year survival rate near 50%, ovarian cancer has the highest mortality rate of any gynecologic malignancy (5). Epithelial ovarian cancer (EOC) comprises 90% of cases, with OCCC a rare subtype marked by poor prognosis. Genetically distinct, featuring ARID1A inactivation, higher PIK3CA/PTEN and lower p53 and BRCA1/2 mutation rates, OCCC is intrinsically chemoresistant (6). Despite chemotherapy response rates between 10-25% and 6-8% in recurrent disease, treatment standards mirror other EOC subtypes consisting of cytoreductive surgery and platinum-based systemic therapy (6, 7). Novel treatments are warranted and interest has grown in immune-checkpoint inhibitors (ICI), angiogenesis targets, and ARID1A synthetic lethal interactions (8).

### Lenvatinib and pembrolizumab

Bevacizumab, the first vascular endothelial growth factor (VEGF) inhibitor approved for ovarian cancer, improved progression-free survival (PFS) when added to chemotherapy in newly diagnosed patients on ICON7 and GOG 0218, and recurrent disease on AURELIA (9–11). While no overall survival (OS) benefit was seen, chemoresistant histologies showed the greatest benefit, highlighting the potential of antiangiogenic agents (12, 13).

Lenvatinib, a multikinase inhibitor including VEGF, demonstrated a 71% objective response rate (ORR) in patients with platinum-resistant ovarian cancer in phase 1 data (14). Pre-clinical studies show enhanced tumor suppression when combined with anti-PD-1 agents such as pembrolizumab (15). Pembrolizumab alone showed modest activity in recurrent ovarian cancer in KEYNOTE-100, and combination lenvatinib/pembrolizumab has demonstrated improved PFS and OS in renal cell carcinoma (CLEAR) and endometrial cancer (KEYNOTE-775) (16–18). Likewise, case reports have show durable responses to combination therapy in recurrent OCCC (19, 20). While not published at the time of treatment, the LEAP-005 phase II trial evaluating lenvatinib/pembrolizumab in previously treated advanced ovarian cancer reported an ORR of 26%- 35%, median PFS of 6.2 months, and OS of 21.3 months, supporting use as fourth-line therapy (21). Further trials, such as the external trial this patient attempted enrollment, remain ongoing.

The clinical course of this patient reflects the established regimens above, with systemic response aside from her progressive liver metastasis. To date her PFS and clinicopathologic response support lenvatinib/pembrolizumab combination therapy and exceed that of LEAP-005. Factors related to this could reflect her unique biology, limited statistical sample size, or the impact of adding radiation with SFRT.

### Spatially fractionated radiation therapy

For metastatic or unresectable disease, stereotactic body radiation (SBRT) delivering high doses of radiation provides symptom palliation, reduces disease burden, and may extend survival. For tumors greater than 5 cm however, concern about unacceptable toxicity exists (22). Spatially fractionated radiation therapy (SFRT or “GRID”) allows for dose escalation in these cases, creating a heterogenous dose distribution of high dose “peaks” with intervening low dose “valleys” (23). Reports of significant symptom relief, tumor regression, and disease control in historically radio-resistant histologies have been documented, including local bystander and distant abscopal effects (24).

Despite being cited as experimental for this patient, SFRT use dates back to 1909, utilizing physical blocks with holes in a “GRID” pattern to modulate dose and treat large superficial tumors (25). While not novel, modern technology and techniques such as VMAT have expanded applicability to deep-seated tumors or those abutting OARs. Enhanced understanding of the tumor microenvironment (TME) and radiobiology have further renewed interest in SFRT. Intratumor hypoxia, present in bulky tumors, may increase hypoxia-inducible factors resulting in angiogenesis, cancer stem cell survival, immune evasion, and radiation resistance (26–28). Dose-dependent responses to radiation may overcome these barriers and have shown immunomodulatory effects. In addition to direct cytotoxic effects, high-dose radiation may promote immunogenic cell death and enhance immune activation via cytokine and neo-antigen release and T-cell priming; upregulating PD-L1 and major histocompatibility class 1 expression, and activating the cGAS-STING pathway to promote immune cell maturation and polization (29). Low-dose radiation may reshape the TME by polarizing macrophages to a M1 phenotype, increasing TNF-alpha and IL-12, and promoting normalization of tumor vasculature to improve oxygenation and immune infiltration (30). In the case of SFRT, combination high and low dose radiation may therefore synergistically augment anti-tumor response and microenvironment reprogramming, with postulation that intervening low dose regions may preserve perfusion for circulating factors involved in anti-tumor immunity (24, 31).

As ICI efficacy relies on amplifying existing T-cell response, these radiation immunomodulatory effects may provide a synergistic approach to tumor control, and case reports utilizing SFRT with ICIs have shown promising results with partial and complete responses (24, 25). Additionally, re-sensitization following SFRT in pembrolizumab refractory patients has been reported (32). While this patient did not receive immunotherapy concurrently with SFRT, her complete pathologic response and greater than expected PFS may reflect the downstream impact of radiation, potentiating the anti-angiogenesis properties of lenvatinib and immunological response to pembrolizumab. Further investigation of this interaction is ongoing and could support future multimodal management and insurance coverage of SFRT.

### Clinical trials, expanded access, and right to try

Drug development and new technology implementation are largely achieved through clinical trials. These trials offer hope and are pivotal in advancing cancer care, but remain plagued by long accrual times, inequitable access, and homogenous representation, raising concern about generalizability (3). Patient-level barriers arise from logistics being away from home, travel costs, job concerns, and varying degrees of social support. Additional restrictions arise from provider lack of awareness and bias, study and institutional requirements, and resource availability (3, 33). As this case demonstrated, local trial availability may be limited, forcing patients to travel to receive care; 38-52% of patients commute over an hour to participate on clinical trials - even longer in the Central United States (34). Despite seeking out care and meeting initial enrollment criteria, patients may be disqualified due to stringent inclusion/exclusion criteria, historically included for statistical power, inadvertently result in generalizability uncertainty. The trial herein highlights these disparities, with exclusion criteria listing “those who’s social and economic status limit compliance with study requirements” – a stipulation not uncommon in trials (35).

To address these issues the FDA Oncology Center of Excellence (OCE) launched Project Equity, a health initiative aimed at addressing disparities in cancer trials, and multiple organizations have arisen to bolster patient engagement (3, 36). The Affordable Care Act (ACA) additionally requires coverage of routine costs for patients on trials, but does not address indirect costs such as travel, lodging, and lost wages being away from work (33). Certain Medicaid plans offer lodging assistance, and non-profit organizations such as the American Cancer Society and Ronald McDonald house provide short term housing options (37). Additionally, local transit agencies, volunteers, and church groups may offer assistance. However, resources may not be universally available, and as our patient experienced, navigating them can be time-consuming. Early and robust social work involvement may help connect patients with the right resources— assuming availability.

For patients’ ineligible for trials, the FDA Expanded Access (EA) pathway provides a route to pursue agents off trial. Known as “compassionate use”, patients with serious or life-threatening illnesses may utilize the EA pathway to gain access to investigational drugs, biologic, or medical devices when no comparable alternatives are available (38). Assessed on a case-by-case basis, this pathway may be cumbersome and resource intensive, requiring coordination between the FDA, IRB, patient, physician, drug company, pharmacy, billing, and more. To assist, the FDA OCE launched Project Facilitate in 2019, a pilot to help providers request access to investigational therapies through the EA pathway (38). The federal Right to Try Act (RTT) was passed in 2018, offering an alternate avenue from EA in obtaining access to investigational drugs, aiming to streamline the process by removing FDA approval and IRB oversight (39). RTT remains restrictive however, limited to terminally ill patients who have exhausted approved treatment options and are ineligible for trials. Investigational agents must have completed a phase I clinical trial and be under continued trial investigation for use or effectiveness (40).

While both the EA and RTT pathways aim to improve patient access, a survey of community oncologists showed that despite awareness, only 46% had attempted to gain access to an investigational drug via EA, 14% via RTT (41). Of those who utilized these pathways, success rates were high, with 89% obtaining access with EA, and 73% with RTT. While highlighting the success this program can have, poor utilization rates reflect the cumbersome processes and the administrative, clinical, and logistical challenges they pose. For patients who do gain access, companies developing the investigational product may only charge patients the direct product cost and shipping. They are not required to approve requests for access, and insurance companies likewise are not required to provide coverage (39). This may render a potentially beneficial treatment unattainable due to financial barriers, despite technical approval, and may be devastating for patients without alternatives.

### Cancer as chronic illness and financial toxicity

Financial toxicity refers to the negative effect cancer treatments and their financial burdens have on patient quality of life, and is associated with early mortality and morbidity (42). As treatments evolve, cancer is often a chronic condition marked by multiple lines of therapy. Chronic illness is the most expensive condition for employers, with rising costs increasingly shifted to patients through insurance premiums, copays, and deductibles (33). Between 1999 and 2018, insurance premiums increased 239%, while wages only increased 68% (43). Within one year of an early-stage cancer diagnosis, patients report losing one-third of their annual income (44). Statistically, patients with cancer are 2.65 times more likely to file for bankruptcy, and 71% more likely to undergo foreclosure, repossession, or mortgage deliquency than those without cancer (2, 45).

Resources exist to offset treatment costs during trials but are generally short-term and poorly equipped to support chronic cancer costs. Policy changes such as the Patient Protection and Affordable Care Act (ACA) in 2010 have attempted to curtail rising costs and increase insurance accessibility and coverage. While this has had some benefit, out-of-pocket (OOP) costs have continued to rise. One analysis found nearly 40% of households lacked liquid resources to meet mid-range OOP limits, and 51% for high-range limits (42, 46). Drug therapies such as chemo- or immunotherapies are major cost drivers, and up to 45% of patients report medication nonadherence to cut costs (47). Patients additionally cut basic needs such as food or clothing, skip doctor visits and tests, or forgoing mental health care (42). States have implemented copay caps and co-pay assistance programs to offset costs. Many have also called for drug transparency, and organizations like the American Medical Association have launched Truth in Rx, a campaign to promote price education and urge legislative action to address drug costs (48).

Despite these efforts, financial toxicity solutions remain limited. Patients remain vulnerable to rising costs and reliant on insurance, public programs, and charity to access care. As treatment grows more complex and prolonged, patients turn to providers to navigate the system – yet fewer than 20% of patients talk about finances with their oncologist (43). Crowdfunding has surged, with cancer accounting for 41.1% of all medical compaigns on GoFundMe (49, 50). Even with approval and support, such as this patient, ongoing treatment remains vulnerable to insurance, or policy changes beyond their control.

This patient’s experience reflects the challenges embedded in her local healthcare system. This does not exist in isolation. Multiple systematic reviews have unveiled the global burden of financial toxicity, with a recent review of 35 articles from 10 countries in 3 continents finding that more than half of all cancer patients experience catastrophic health expenditures (51). The healthcare ecosystem is increasing complex, with multi-faceted costs. Global and regional solutions are urgently needed.

## Conclusion

This patient’s complete clinical and pathologic response to lenvatinib, pembrolizumab, and SFRT adds evidence to support these therapies in OCCC and underscores the promise of multimodal approaches. Further research on the immunomodulatory effects of radiation and SFRT is warranted, as well as the response durability of lenvatinib/pembrolizumab in OCCC. Additionally, her case exemplifies the challenges in accessing innovative treatments. Insurance denials, clinical trial ineligibility, and financial toxicity create significant barriers, forcing patients to navigate a fragmented and opaque system. As cancer shifts to a chronic disease, these issues become even more pressing and far-reaching. Addressing costs, coverage, and providing resource support could help ease this burden, while revisiting restrictive clinical trial eligibility criteria may make life-saving treatments more accessible and studied in “real-life” settings. The growing complexity of cancer care raises important questions about how to balance innovation and access, pushing not only the medical community, but the global healthcare ecosystem and society at-large to consider how new treatments are developed, reimbursed, and delivered to those who need them most.

## Patient perspective

“I am 54 years old, and I would like to offer my perspective on my journey with my battle with Ovarian Clear Cell Carcinoma, and the obstacles I faced with my fight to live. Six months after losing my husband in 2020 to death, and my only child to a genetic mental illness, my journey to live began for me.

In Feb of 2021 I was diagnosed with a rare form of ovarian cancer, clear cell carcinoma. My treatment and my cancer is all documented in the article Mayo has written on my treatment success, and struggles,and that long road that got me here today, but my personal experiences, fighting the disease itself, as well as what I personally had to go through to be able to see this article written, and to be here to offer my perspective is not. First I want to say that I strongly feel that if I had received the lenvatinib and the pembrolizumab in the beginning of my disease, I quite possibly would not have had to endure the fight I had to go through, nor the severe disease progression that I experienced after chemotherapy, the 13 centimeter mass to my liver, and the hypercalcemia in my blood, and months of being very sick with a disease that does not respond to chemotherapy. I knew from doing my own research for months online, but my Doctor also knew the best medicine for me was available at a clinical trial in Boston Massachusetts, and I live in Wisconsin, when we first discussed this I was 85 pounds, and very sick, I lived all alone, and I didnt think I could do it, but then one morning I woke up crying. I said to myself you have no choice, Michelle, you have to go to Boston. So that morning I phoned Mayo Clinic and made an appointment with my Doctor. When I met with him, I said, I’m going to Boston. He was just as excited as me. I had to travel hundreds of miles from my home, I was 85 pounds and very sick, I had no one that could go with me, but I had no other choice, after all of this, I was excluded from that trial due to a pulmonary embolism. I was devastated to say the least, but my wonderful Doctor at Mayo Clinic fought for me to try the medicine off label, this man is, and will always be my hero. The medicine was a miracle for me. I was living to live again, my cancer was all shrinking, hypercalcemia had disappeared, and I gained 20 pounds from the 85 pounds I was when I attempted that clinical trial, but most importantly, I was making cherished memories with my Granddaughter, and the miracle for me was time. Then my employee sponsored insurance changed companies, they said I no longer could have the medicine, they claimed I was experimental investigational, even with all the ct scans that showed substantial shrinkage in my tumors, and all my clinical improvement, they denied even after an appeal, and even with me having congress involved in helping me fight for my medicine. I felt I had nowhere to turn, but I had to try, and I navigated to the top of my employer sponsored insurance, I had been at my job for 30 years, but I was on leave for a long time fighting my cancer, my employer overturned the denial for me, which was yet another miracle, I finally got to resume my medicine. I still have worries to this day that at any time this can be taken from me. It’s hard to think about, and It’s always in the back of your mind, but I know I wont ever give up. I started my journey in 2021, Chemotherapy does not work for my rare form of cancer, and yet it’s 2025, and this treatment is still not approved. I belong to several blogs with people that have my same disease, I see them suffer and pass away all the time, many of whom are younger than me. We have these medicinal miracles, and yet so few get access to them in time. I hope that my struggle, my miracles, and my story can help change the lives of others, and that they can get the medication I have, and have time to make the memories that I have been fortunate to be able to make. My team at Mayo Clinic, and the fight I have to never give up that has made it all possible for me.”

## Data Availability

The original contributions presented in the study are included in the article/supplementary material. Further inquiries can be directed to the corresponding author.
